# Asbestos

**Published:** 1896-04

**Authors:** 


### Asbestos.

A novel surgical dressing is advocated by Dr. Kane in the use of asbestos, in its various forms.

It is claimed for this substance that it is soft, non-irritating, possessing absorbent qualities superior to cotton and has the great advantage of sterilization without elaborate apparatus.

Dr. Kane is accustomed to carry it in his surgical case and to sterilize it at the time of the operation by throwing it into a fire and allowing it to attain its characteristic cherry color by the heat of the coals.

He also uses the same dressing repeatedly by this method of sterilization. If all is true that is claimed for this material, it possesses undoubted value.—*Atlantic Med. Weekly.*

